# Dataset for transcriptome analysis of abscisic acid degrading bacterium *Novosphingobium* sp. P6W

**DOI:** 10.1016/j.dib.2019.105001

**Published:** 2019-12-16

**Authors:** Natalia E. Gogoleva, Tatiana A. Konnova, Timur T. Ismailov, Alexander S. Balkin, Andrey A. Belimov, Yuri V. Gogolev

**Affiliations:** a Kazan Institute of Biochemistry and Biophysics, Kazan Scientific Center of RAS, 2/31 Lobachevsky St., Kazan 420111, Russian Federation; b Institute of Fundamental Medicine and Biology, Kazan (Volga Region) Federal University, 18 Lenina St., Kazan 420021, Russian Federation; c Center of Shared Scientific Equipment “Persistence of Microorganisms”, Institute for Cellular and Intracellular Symbiosis, Ural Branch of Russian Academy of Sciences, Orenburg, Russian Federation; d All-Russia Research Institute for Agricultural Microbiology, 3 Sh. Podbelskogo St., Saint Petersburg 196608, Russian Federation

**Keywords:** Plant growth-promoting rhizobacteria (PGPR), *Novosphingobium* sp., RNA-seq, Illumina, Rhizosphere

## Abstract

Plant growth-promoting rhizobacteria (PGPR) improve plant productivity and stress resistance. The mechanisms involved in plant-microbe interactions include the modulation of plant hormone status. The *Novosphingobium* sp. strain P6W was previously described as the bacterium capable of abscisic acid (ABA) degradation, and its inoculation decreased ABA concentrations in planta. The metabolic pathway for the ABA degradation in bacteria is still unknown. Here we present transcriptome data of *Novosphingobium* sp. P6W grown in the medium supplemented with ABA or fructose as the carbon source. Cleaned FASTQ files for the RNA-seq libraries are deposited in the NCBI Sequence Read Archive (SRA, Identifier: SRP189498) and have been assigned BioProject accession PRJNA529223.

**Table:** Specifications Table

Subject	Biology
Specific subject area	Transcriptomics
Type of data	Transcriptome sequences, table, figure
How data were acquired	High-throughput RNA-sequencing with Illumina HiSeq 2500
Data format	Clean data, FASTQ
Experimental factors	Growth of soil bacteria in a minimal medium supplemented with ABA
Experimental features	Datasets for bacterial cultures utilizing ABA or fructose and under carbon starvation conditions
Data source location	Kazan Scientific Centre of RAS, Kazan, Russia.
Data accessibility	Cleaned FASTQ files are deposited in a public repository: Repository name: NCBI SRA Data identification number: PRJNA529223 Direct URL to data: https://www.ncbi.nlm.nih.gov/bioproject/529223

**Table:** 

**Value of the Data** • These datasets will be valuable to the PGPR research community for characterizing changes in rhizobacterial gene expression caused by phytohormones and depending on environmental conditions. • Downstream analysis will allow the identification of genes involved in bacterial ABA degradation. • Cleaned sequencing reads can be further processed by researchers using their own bioinformatic algorithms and analyzed together with their own data.

## 1 Data description

**Fig. 1:**
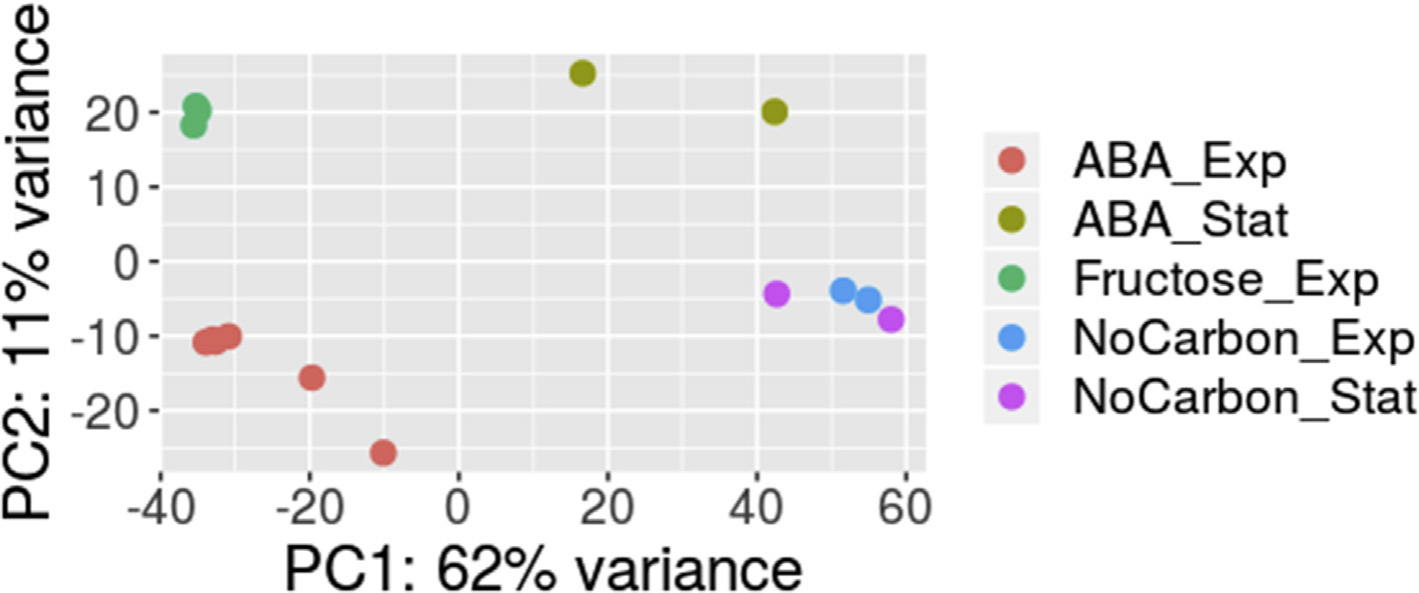
Principal component analysis (PCA) of the general transcriptome characteristics. The first principal component (component 1) accounted for 62% and the second principal component (component 2) for 11% of the total variance in the dataset. Legend description: “ABA_exp”and “ABA_Stat” – samples of cultures grown in ABA supplemented medium taken at the exponential and stationary phases respectively (see samples ABA 1–6 and ABA 7 and 8 in Table 1); “Fructose_Exp” – samples of exponential phase cultures grown in the medium supplemented with fructose (see samples Fructose 1–3 in Table 1); “NoCarbon_Exp”and “NoCarbon_Stat” – samples of cultures incubated under carbon starvation for 24 and 48 hours respectively (see samples NoCarbon 1 and 2 and NoCarbon 3 and 4 in Table 1).

The dataset contains cleaned sequencing data obtained through the transcriptome sequencing of *Novosphingobium* sp. P6W grown in the medium supplemented with ABA or fructose as the sole carbon source and under carbon starvation conditions. Samples for transcriptome profiling were collected at the exponential and stationary growth phases. Cleaned FASTQ files were deposited in NCBI Sequence Read Archive and accessible through the BioProject PRJNA529223. Information about bacterial culture samples is presented in Table 1. Reads were mapped onto the reference genome sequence and the coverage data were obtained. Statistics of sequence reads and sequence coverage data are shown in Table 2. PCA plot of RNA-seq data presented in Fig. 1 demonstrates the variance between sample groups and sample replicates according to gene expression levels. Each dot in the Fig. 1 indicates particular sample.

**Table 1:** Samples of the *Novosphingobium* sp P6W cultures.

Sample name	Biological replicates	Carbone source	Duration of cultivation, hours	Culture density, OD	Accession number
ABA exponential phase	ABA_1	ABA	24	0.23	SRX5577386
	ABA_2	ABA	24	0.21	SRX5577385
	ABA_3	ABA	24	0.21	SRX5577384
	ABA_4	ABA	24	0.24	SRX5577383
	ABA_5	ABA	24	0.21	SRX5577391
	ABA_6	ABA	24	0.20	SRX5577381
ABA stationary phase	ABA_7	ABA	48	0.55	SRX5577382
	ABA_8	ABA	48	0.51	SRX5577380
Carbon starvation exponential phase	NoCarbon_1	absent	24	0.13	SRX5577387
	NoCarbon_2	absent	24	0.10	SRX5577392
Carbon starvation stationary phase	NoCarbon_3	absent	48	0.16	SRX5577379
	NoCarbon_4	absent	48	0.19	SRX5577378
Fructose exponential phase	Fructose_1	fructose	18	0.25	SRX5577390
	Fructose_2	fructose	18	0.28	SRX5577389
	Fructose_4	fructose	18	0.25	SRX5577388

**Table 2:** Cleaned reads and reads mapped on reference genome.

Library	Number of cleaned reads	Number of reads mapped on genome	% Mapped reads
ABA_1	10,899,064	10,346,749	94.93
ABA_2	10,757,369	10,281,619	95.58
ABA_3	9,060,795	8,713,460	96.17
ABA_4	12,313,428	11,778,892	95.66
ABA_5	9,715,928	9,659,951	99.42
ABA_6	11,740,625	10,636,562	90.60
ABA_7	12,473,706	12,413,817	99.52
ABA_8	6,292,959	5,820,562	92.49
NoCarbon_1	9,325,126	9,184,277	98.49
NoCarbon_2	4,655,901	4,254,299	91.37
NoCarbon_3	6,234,953	5,123,816	82.18
NoCarbon_4	4,468,833	4,286,867	95.93
Fructose_1	12,282,002	11,014,354	89.68
Fructose_2	10,869,930	9,944,951	91.49
Fructose_4	12,513,546	10,247,348	81.89

## 2 Experimental design, materials, and methods

### 2.1 Bacterial strains and growth conditions

The *Novosphingobium* sp. P6W strain was initially isolated from the rhizosphere of rice (*Oryza sativa* L.) seedlings [1]. Complete genome sequencing for this strain was performed previously [2]. Bacterial cells were grown aerobically at 28 °C in a minimal medium (g L-1: MgSO4x7H2O - 0.3; NH4NO3 - 0.5; KH2PO4 - 1.36; FeCl3 - 0.002; pH 6.7) supplemented with 250 mg/L (±)-abscisic acid (Sigma) or 250 mg/L d-fructose (Sigma) as a sole carbon source.

### 2.2 Experiment design

To identify the genes involved in ABA metabolism, the transcriptome profiles of exponential phase cultures growing in the minimal medium supplemented with ABA or fructose were compared. To exclude genes associated with stress adaptation, samples of cultures incubated under carbon starvation conditions for 24 and 48 hours were taken as corresponding controls. It was important to obtain information about the genes that decrease activity at the substrate depletion. For this purpose, samples of cultures grown in the ABA supplemented medium at the stationary phase were also taken.

### 2.3 Library construction and sequencing

Bacterial cultures were fixed with an equal volume of cold RNA-stabilizing solution (19% ethanol, 1% acidic phenol, pH 5.5) on ice for 30 minutes. Cells were harvested by centrifugation and RNA isolation was performed using RNA Extract Reagent (Evrogen, Russia) according to the manufacturer's protocol. DNA contaminants were removed using RNase-free DNase I kit (Ambion, USA). The integrity of the RNA was checked by Agilent 2100 bioanalyzer (USA). For rRNA removal the Ribo-Zero kit for Gram-negative bacteria (Illumina, USA) was used.

NEBNext Ultra Directional RNA Library Prep Kit for Illumina was used to prepare RNA-seq libraries. The resulting average size of the cDNA libraries was approximately 300 bp. Libraries were sequenced using the Illumina HiSeq 2500 sequencing platform.

### 2.4 Sequence QC and filtering

144,262,494 reads were obtained in total with a length of 60 nucleotides (Table 1). FastQC software (Version 0.11.5) [3] was used to assess the quality of the raw Fastq files and clean reads. Raw reads were filtered using BBDuk (v. 37.23, http://jgi.doe.gov/data-and-tools/bb-tools/) to remove Illumina adapters, NEB indexes and to quality-trim right end to Q20 (ktrim = r k = 23 mink = 11 hdist = 1 tpe tbo minlen = 25 qtrim = r trimq = 20). Thereafter, the rRNA reads were eliminated by using SortMeRNA v2.1 program [4].

### 2.5 Reads alignment to the reference genome

The high-quality reads were mapped onto the genome sequence of the *Novosphingobium* sp. P6W strain (assembly: GCA_000876675.2) (ftp://ftp.ncbi.nlm.nih.gov/genomes/all/GCF/000/876/675/GCF_000876675.2_ASM87667v2/GCF_000876675.2_ASM87667v2_genomic.fna.gz). HISAT2 version 2.1.0 [5] was used to build index of reference genome and align clean reads to reference genome with the following parameters: hisat2 -p --dta -x -U -S. SAM files of alignments created by HISAT2 were converted to BAM files using SAM-tools view [6]. Coverage estimates and reads mapping statistics are presented in Table 2. DESeq2 [7] was used to assess variance between sample groups and sample replicates using principle component analysis (PCA). PCA plot shown in the Fig. 1 demonstrates the overall quality of our sample collection, library preparation, and sequencing.

## References

[1] Belimov A.A., Dodd I.C., Safronova V.I., Dumova V.A., Shaposhnikov A.I., Ladatko A.G., Davies W.J. (2014). Abscisic acid metabolizing rhizobacteria decrease ABA concentrations in planta and alter plant growth. Plant Physiol. Biochem..

[2] Gogoleva N.E., Nikolaichik Y.A., Ismailov T.T., Gorshkov V.Y., Safronova V.I., Belimov A.A., Gogolev Y.V. (2019). Complete genome sequence of the abscisic acid-utilizing strain *Novosphingobium* sp. P6W. 3 Biotech.

[3] Andrews S. (2010). FastQC: a quality control tool for high throughput sequence data. http://www.bioinformatics.babraham.ac.uk/projects/fastqc/.

[4] Kopylova E., Noé L., Touzet H. (2012). SortMeRNA: fast and accurate filtering of ribosomal RNAs in metatranscriptomic data. Bioinformatics.

[5] Kim D., Langmead B., Salzberg S.L. (2015). HISAT: a fast spliced aligner with low memory requirements. Nat. Methods.

[6] Li H., Handsaker B., Wysoker A., Fennell T., Ruan J., Homer N., Marth G., Abecasis G., Durbin R., 1000 Genome Project Data Processing Subgroup (2009). The sequence alignment/map format and SAMtools. Bioinformatics.

[7] Love M.I., Huber W., Anders S. (2014). Moderated estimation of fold change and dispersion for RNA-seq data with DESeq2. Genome Biol..

